# Ocular administration of brinzolamide leading to Stevens-Johnson syndrome/toxic epidermal necrolysis overlap: A case report and review

**DOI:** 10.1097/MD.0000000000046362

**Published:** 2026-05-12

**Authors:** Huayi Lu, Wenqiang Xu, Yali Wu, Mingrui Zhang, Sen Ma

**Affiliations:** a Department of Ophthalmology, The First Affiliated Hospital of USTC, Division of Life Sciences and Medicine, University of Science and Technology of China, Hefei, Anhui Province, China; b Discipline Planning Management Office, The First Affiliated Hospital of USTC, Division of Life Sciences and Medicine, University of Science and Technology of China, Hefei, Anhui Province, China; c Department of Ophthalmology, Leiden University Medical Center (LUMC), Leiden, The Netherlands.

**Keywords:** brinzolamide, carbonic anhydrase inhibitor, ophthalmic drug reaction, Stevens-Johnson syndrome, toxic epidermal necrolysis

## Abstract

**Rationale:**

Stevens-Johnson syndrome (SJS) and toxic epidermal necrolysis (TEN) are severe, life-threatening hypersensitivity reactions most often induced by systemic medications. Reports of SJS/TEN caused by topical ophthalmic sulfonamide-derived carbonic anhydrase inhibitors, such as brinzolamide, are extremely rare.

**Patient concerns:**

A 31-year-old man underwent pars plana vitrectomy for traumatic retinal detachment in the right eye and subsequently developed secondary glaucoma. He was treated with topical brinzolamide 1% twice daily. On day 6 of treatment, he developed high-grade fever and erythema on the upper limbs, which rapidly progressed to widespread edematous erythema, bullae, epidermal detachment, and erosions involving 99% of the total body surface area. Ocular involvement was asymmetric: the right eye showed eyelid ulceration and conjunctival hyperemia, while the left eye had more severe manifestations, including marked conjunctival chemosis and extensive corneal epithelial sloughing.

**Diagnosis:**

Laboratory tests revealed leukopenia, elevated C-reactive protein, abnormal liver enzymes, and hypoalbuminemia. Blood cultures grew *Escherichia coli* and *Streptococcus* species. Causality assessment demonstrated a Naranjo score of 7 and an ALDEN score of 5, indicating a probable relationship between brinzolamide and SJS/TEN overlap syndrome.

**Interventions:**

The patient was treated with intravenous immunoglobulin, systemic corticosteroids, broad-spectrum antibiotics, antifungal therapy, supportive care, and ocular surface protection.

**Outcomes:**

Over 4 weeks, cutaneous lesions resolved with residual hyperpigmentation. The left eye developed symblepharon and dense corneal scarring, requiring penetrating keratoplasty, resulting in postoperative visual acuity improvement to 0.05. The right eye remained without light perception due to preexisting retinal detachment.

**Lessons:**

Topical ocular administration of sulfonamide-based drugs may trigger life-threatening systemic hypersensitivity reactions. Thorough allergy history and human leukocyte antigen genotyping should be considered before prescribing. Early recognition of prodromal symptoms, particularly ocular signs, and prompt multidisciplinary intervention are critical to preventing severe complications and irreversible vision loss.

## 1. Introduction

Carbonic anhydrase inhibitors (CAIs) are widely used in the treatment of glaucoma and ocular hypertension, primarily by reducing aqueous humor production to lower intraocular pressure. Among them, brinzolamide and dorzolamide are commonly formulated as topical ophthalmic agents, with generally favorable safety profiles and good ocular tolerability. However, despite their low systemic bioavailability (<5%), potentially life-threatening adverse reactions may still occur in genetically susceptible individuals.^[1,2]^

Stevens-Johnson syndrome (SJS) and toxic epidermal necrolysis (TEN) are severe cutaneous adverse drug reactions characterized by widespread epidermal necrosis and mucosal involvement, and are primarily mediated by type IV (delayed-type) hypersensitivity reactions involving cytotoxic T lymphocytes and proinflammatory cytokines. Based on the body surface area (BSA) affected by epidermal detachment, cases are classified as SJS (<10% of BSA), SJS/TEN overlap (10%–30%), and TEN (>30%).^[3,4]^

To date, most studies on SJS/TEN have focused on reactions to systemic medications, with only extremely rare reports implicating topical CAI eye drops as the causative agents. Previous case reports have linked topical sulfonamide-containing drugs such as sulfacetamide with SJS/TEN, but no prior cases have definitively attributed the reaction to brinzolamide monotherapy. Here, we report the documented case of SJS/TEN overlap syndrome induced by topical ocular administration of brinzolamide, and review the potential underlying mechanisms and clinical management strategies.

## 2. Case

A 31-year-old previously healthy male underwent 23-gauge pars plana vitrectomy with wide-angle membrane peeling, intravitreal fluid aspiration, endolaser photocoagulation, fluid-air exchange, perfluorocarbon liquid injection, and silicone oil tamponade for traumatic retinal detachment in his right eye. Postoperatively, he was treated with topical levofloxacin, tobramycin-dexamethasone eye drops, and oral methylprednisolone.

On postoperative day 18, he presented with right eye redness, pain, and elevated intraocular pressure, and was diagnosed with secondary glaucoma (right eye). Steroid eye drops were discontinued, and brinzolamide 1% eye drops were initiated twice daily.

**Figure 1.**
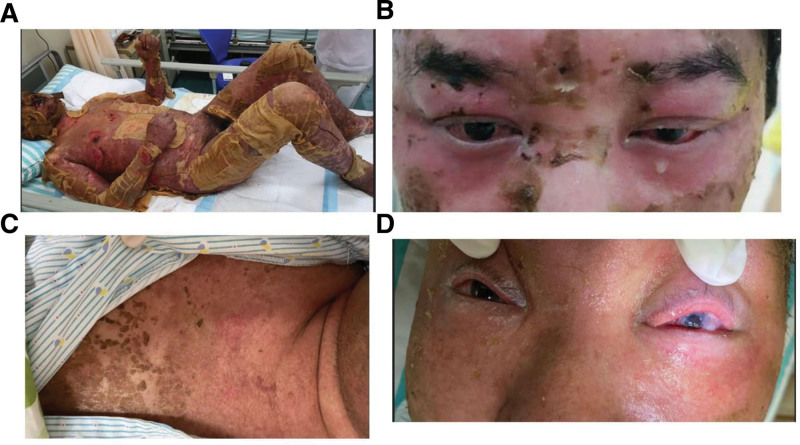
Clinical photographs depicting the acute and recovery phases of SJS/TEN. (A) Extensive erythematous bullae and epidermal detachment involving the trunk and extremities during the acute stage. (B) Ocular involvement is characterized by marked eyelid edema and conjunctival injection. (C) Postinflammatory hyperpigmentation was observed during the recovery phase, with resolution of epidermal lesions. (D) Long-term ocular sequelae, including symblepharon formation and corneal opacity. SJS = Stevens-Johnson syndrome, TEN = toxic epidermal necrolysis.

On day 6 of brinzolamide use, the patient developed a high fever (up to 40°C) and erythematous rashes on both upper limbs, which rapidly progressed. Upon admission, he had widespread edematous erythema, flaccid bullae, and coalescing blisters over his entire body. Mucosal erosions and exudation were present on the lips, eyelids, oral mucosa, and perineum. The initial area of epidermal detachment was 20%, progressing to over 80% within 24 hours. Nikolsky sign was positive (Fig. 1A), indicating skin fragility.

Acute-phase ocular findings were significant and asymmetric. The right eye (treated eye) showed eyelid margin ulceration, conjunctival hyperemia, and partial meibomian gland orifice obstruction; the left eye exhibited more severe conjunctival chemosis and extensive corneal epithelial detachment, accompanied by severe pain that precluded slit-lamp examination (Fig. 1B). Despite the right eye being the site of brinzolamide application, the left eye suffered more extensive ocular surface damage, possibly due to local tissue susceptibility or immunologic factors. Due to disease severity, only external ocular photographs were preserved.

Laboratory findings revealed leukopenia (3.78 × 10^9^/L), elevated neutrophil ratio (85.2%), reduced lymphocyte ratio (9.9%), thrombocytopenia (91 × 10^9^/L), and elevated inflammatory markers (CRP: 156.06 mg/L; high-sensitivity CRP: >5.00 mg/L). Liver enzymes were elevated (ALT: 57.2 IU/L, AST: 49.7 IU/L, γ-GGT: 92.5 IU/L), with hypoalbuminemia (34.4 g/L). Blood cultures were positive for *E coli* and hemolytic *Streptococcus* species; wound fluid cultures identified *Staphylococcus aureus*, *E coli*, and *Candida parapsilosis*. Serum(1-3)-β-D-glucan was significantly elevated. Stool Gram stain revealed 70% Gram-negative bacilli (Table 1). The SCORTEN score was 2, corresponding to an estimated mortality of 12.1%. The Naranjo score was 7, and the ALDEN score was 5, both indicating a “probable” causal relationship between brinzolamide and the adverse reaction (Tables 2 and 3).^[5,6]^

**Table 1:** Laboratory results on admission are indicative of systemic inflammation in SJS/TEN.

Variable	Reference range, adults	On admission
Blood routine		
White-cell count (×10^9^/L)	4.0–10.0	3.78
Neutrophils (%)	40–75	85.2
Lymphocytes (%)	20–50	9.9
Monocytes (%)	2–8	3.5
Red-cell count (×10^12^/L)	4.0–5.5	5.14
Hemoglobin (g/L)	130–175	154
Platelet count (×10^9^/L)	150–400	91
Inflammatory markers		
C-reactive protein (CRP, mg/L)	0–5	156.06
High-sensitivity CRP (mg/L)	0–10	>5.00
(1-3)-β-D-glucan (pg/mL)	<100	291.67 (August 24); 38.95 (September 2)
Blood culture	—	*E coli*, *Streptococcus* positive
Electrolytes		
Potassium (mmol/L)	3.5–5.5	3.99
Sodium (mmol/L)	135–145	131.03
Chloride (mmol/L)	98–108	91.58
Liver function		
Alanine aminotransferase (ALT, IU/L)	7–40	57.2
Aspartate aminotransferase (AST, IU/L)	13–40	49.7
Alkaline phosphatase (ALP, IU/L)	40–150	74.8
Gamma-glutamyl transferase (GGT, IU/L)	7–50	92.5
Albumin (g/L)	35–55	34.4

ALT = alanine aminotransferase, AST = aspartate aminotransferase, CRP = C-reactive protein, GGT = gamma-glutamyl transferase.

**Table 2:** Naranjo causality assessment table.

Question	Yes	No	Not known or not done
1. Are there previous conclusive reports on this reaction?	+1		
2. Did the adverse event appear after the suspected drug was administered?	+2		
3. Did the adverse reaction improve when the drug was discontinued or a specific antagonist was given?	+1		
4. Did the adverse reaction reappear when the drug was readministered?			0
5. Are there alternative causes that could have caused the reaction?		+2	
6. Did the reaction reappear when a placebo was given?			0
7. Was the drug detected in the blood (or other fluids) in concentrations known to be toxic?			0
8. Was the reaction more severe when the dose was increased or less severe when the dose was decreased?			0
9. Did the patient have a similar reaction to the same or similar drugs in any previous exposure?		0	
10. Was the adverse event confirmed by any objective evidence?	1		
Total score	**7**		

**Table 3:** ALDEN algorithm criteria and scoring for drug causality.

Criteria	Possible score	Score
Time lag between initial drug intake and to onset of reaction (index day)	−3 to +3	+3
Presence of the drug in the body on the index day	−3 to 0	0
Prechallenge/rechallenge outcome with the suspect drug	−2 to +4	0
Outcome of rechallenge	−2 to 0	0
Drug notoriety for causing SJS/TEN	−1 to +3	+2
Other possible etiologic alternatives	−1, if applicable	0
Total score	5	

A total score of ≥6 is categorized as very probable, 4 to 5 as probable, 2 to 3 as possible, 0 to 1 as unlikely, and <0 as very unlikely. Specifics of the scoring system for each criterion are not described here but can be found in Sassolas et al.

ALDEN = algorithm of drug causality for epidermal necrolysis, SJS/TEN = Stevens-Johnson syndrome/toxic epidermal necrolysis.

Based on clinical presentation (fever, widespread blistering and epidermal detachment, multisite mucosal involvement), a positive Nikolsky sign, histopathology showing full-thickness epidermal necrosis, and BSA involvement >30%, a diagnosis of SJS/TEN overlap syndrome was confirmed. Given the recent drug history and exclusion of other causes, brinzolamide was considered the likely culprit.

The patient received a multidisciplinary treatment regimen, including intravenous immunoglobulin (2 g/kg for 5 days), methylprednisolone (80 mg/day), vancomycin plus imipenem, voriconazole, albumin infusion, electrolyte replacement, wound care, and amniotic membrane transplantation.

### 2.1. Outcome

**Figure 2.**
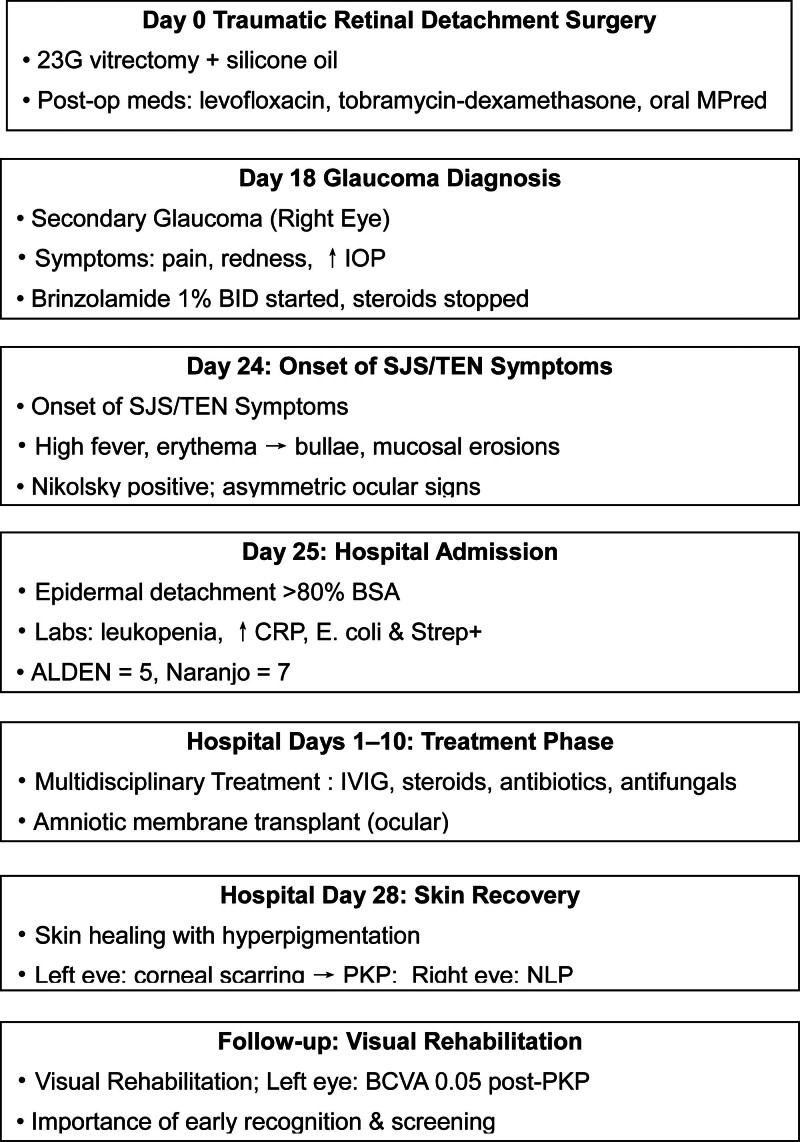
Clinical timeline of the patient with brinzolamide-induced SJS/TEN overlap. Key clinical events, including surgery, drug initiation, symptom onset, hospitalization, treatment course, and outcomes, are shown in chronological order. ALDEN = algorithm of drug causality for epidermal necrolysis, BSA = body surface area, CRP = C-reactive protein, IVIG = intravenous immunoglobulin, NLP = no light perception, PKP = penetrating keratoplasty, SJS = Stevens-Johnson syndrome, TEN = toxic epidermal necrolysis.

By week 4, the skin lesions had largely healed, with residual hyperpigmentation and scarring. Ocular sequelae included symblepharon and corneal scarring in the left eye (Fig. 1C and D), requiring penetrating keratoplasty, after which visual acuity improved to 0.05. The right eye remained without light perception due to prior retinal detachment. In Figure 2, the flow chart describes the clinical timeline of the patient with brinzolamide-induced SJS/TEN overlap.

## 3. Discussion

This case highlights that even topically administered sulfonamide-based medications may induce severe immune-mediated reactions such as SJS and TEN. Brinzolamide, a CAI containing a sulfonamide moiety, may be metabolized into intermediates that form hapten–human leukocyte antigen (HLA) complexes, subsequently activating CD8⁺ T cells and triggering widespread keratinocyte apoptosis.^[7,8]^ Although HLA genotyping was not performed in this patient, prior studies have demonstrated strong associations between specific HLA alleles (e.g., HLA-B15:02, HLA-C06:02) and increased susceptibility to sulfonamide-induced severe cutaneous adverse reactions in Asian populations, suggesting a role for genetic predisposition.^[9,10]^

Despite the low systemic absorption of ophthalmic solutions, the conjunctiva and nasal mucosa represent alternative absorption pathways that may suffice to induce systemic immune responses in highly sensitized individuals.^[11]^ Previous reports have linked other topical sulfonamide eye drops, such as sulfacetamide, to the development of SJS/TEN, emphasizing the need not to underestimate systemic risks from local exposure.^[12]^

SJS/TEN is a life-threatening disease, with a mortality rate of up to 30%. Early combination therapy with intravenous immunoglobulin and systemic corticosteroids has been shown to improve outcomes. Ocular involvement occurs in nearly 100% of SJS/TEN cases and may range from mild conjunctival injection to full-thickness ocular surface sloughing. This can lead to limbal stem cell deficiency, corneal neovascularization, persistent epithelial defects, and eventually blindness.^[2,12]^ Notably, the severity of ocular damage does not necessarily correlate with the extent of cutaneous involvement. Some studies suggest a possible role of HLA class I alleles in determining the severity of ocular complications.

In our patient, the left eye, despite not being the treated eye, exhibited more severe inflammation and tissue damage than the right. This asymmetric ocular involvement suggests that immune-mediated injury in SJS/TEN is not solely dependent on direct local exposure but may be influenced by factors such as ocular surface barrier integrity, local inflammatory microenvironment, and differential immune tolerance. It underscores that SJS/TEN is fundamentally a systemic immunological condition in which target organ damage does not always mirror the route of drug administration.

Although multidisciplinary treatment successfully controlled the systemic inflammatory process in this case, irreversible ocular damage resulted in permanent vision loss. This underscores the need for pretreatment allergy screening, including thorough history-taking and consideration of HLA genotyping when appropriate. Measures such as punctal occlusion during administration may reduce systemic absorption through the nasolacrimal duct. Prompt cessation of suspected agents and early ophthalmologic evaluation are essential to minimize long-term complications.

Furthermore, ophthalmic management should be tailored to both acute and chronic phases of SJS/TEN. During the acute phase, preserving ocular surface integrity through frequent lubrication, prophylactic topical antibiotics, corticosteroids, and early use of amniotic membrane transplantation can mitigate severe complications. In the chronic phase, interventions such as symblepharon lysis, fornix reconstruction, limbal stem cell transplantation, and keratoplasty may be required to restore ocular function and improve visual outcomes.^[2]^

## 4. Conclusion

This report presents a rare case of SJS/TEN overlap induced by topical brinzolamide monotherapy, demonstrating that even localized ophthalmic medications can provoke serious, potentially fatal systemic immune reactions. Based on the clinical insights gained from this case, we propose the following recommendations:

1. Conduct a thorough allergy history before prescribing sulfonamide-containing eye drops and consider HLA genotyping (e.g., HLA-B*15:02) in high-risk individuals, particularly in Asian populations.
2. Enhance pharmacovigilance systems and adverse event reporting for topical sulfonamide agents to better capture rare but severe reactions.
3. Apply punctal occlusion after instillation to reduce systemic absorption via the nasolacrimal duct, and discontinue the medication promptly at the first sign of adverse effects.
4. Initiate multidisciplinary management early for confirmed or suspected SJS/TEN cases, including immunosuppressive therapy, infection control, and ocular surface protection strategies.
5. Strengthen patient education to improve awareness of early hypersensitivity symptoms – especially ocular signs – and encourage timely medical consultation.

## Author contributions

**Conceptualization**: Huayi Lu.

**Data curation**: Huayi Lu, Wenqiang Xu, Yali Wu, Mingrui Zhang.

**Formal analysis**: Wenqiang Xu, Yali Wu, Mingrui Zhang.

**Supervision**: Sen Ma.

**Writing – original draft**: Huayi Lu, Sen Ma.

**Writing – review & editing**: Sen Ma.
